# MST in the wild: Optimizing the mnemonic similarity task for use in diverse environments

**DOI:** 10.1016/j.neuropsychologia.2025.109341

**Published:** 2025-12-03

**Authors:** Lilian Azer, Casey R. Vanderlip, Lizabeth L. Mayer, Luke Ehlert, David Sultzer, Hye-Won Shin, Craig E.L. Stark

**Affiliations:** a Department of Neurobiology & Behavior, University of California, Irvine, University of California Irvine, 1400 Biological Sciences III, Irvine, CA, 92697, USA; b Institute for Memory Impairments and Neurological Disorders, University of California, Irvine, 2642 Biological Sciences III, Irvine, CA, 92697, USA; c Department of Psychiatry & Human Behavior, UCI School of Medicine, 1001 Health Sciences Road, Irvine, CA, 92697, USA; d Somang Society, 5836 Corporate Ave., Ste 110, Cypress, CA, 90630, USA

**Keywords:** Aging, Cognition, Assessment, Cognitive screening, Alzheimer’s disease

## Abstract

**Background:**

Clear guidelines and tools for reliable measures of cognitive decline have yet to be established. This may be due to the absence of access to fully automated, self-administered, and scored cognitive screening tools.

**Methods:**

We used the optimized Mnemonic Similarity Task (oMST; Stark et al., 2023), a web computer-based self-administered tool adapted from the MST. The oMST is designed for cognitive screening and population enrichment, offering a superior alternative to traditional neuropsychological tests. We tested the oMST’s reliability, validity, and accessibility across five experiments with 1685 participants.

**Results:**

Lure discrimination was highly correlated between in-person and remote administration. These results were consistent across various testing sites, demonstrating the oMST’s robustness. Importantly, visual acuity did not impact performance.

**Conclusions:**

Our findings establish the oMST as a reliable and accessible tool for cognitive screening across diverse testing environments and administration methods, addressing critical gaps in early screening for cognitive decline.

## 1. Background

The risk of dementia among middle-aged adults in America is approximately 42 % and it is projected to increase from approximately 514,000 Americans estimated in 2020 to approximately 1 million Americans in 2060 (Fang et al., 2025). Many factors are considered when understanding the risk of dementia such as, sociodemographic factors (i.e., age, race/ethnicity, etc.), APOE ε4 carrier status, and lifestyle and health (Cosentino et al., 2008; Fang et al., 2025; Röhr et al., 2022; Vanderlip et al., 2024). However, factors such as perspectives on cognitive screening, accessibility to cognitive screening measures, and the need for automated, self-administered, and scored screening measures are often overlooked. For instance, the US Preventive Services Task Force (USPSTF (Moyer & U.S. Preventive Services Task Force, 2014; Patnode et al., 2020),) recommendations and older adults’ attitudes towards cognitive screening are important factors to consider when screening for cognitive impairment (Krohne et al., 2011; Martin et al., 2015; Wong and Jacova, 2018) and providing more accessible screening tools for self-administration. Therefore, the present study aimed to test the reliability, validity, and accessibility of a popular research tool, the Mnemonic Similarity Task (MST (Kirwan and Stark, 2007; S. M. Stark et al., 2019); and it’s optimized variant (oMST (C. E. L. Stark et al., 2023);), for cognitive screening and population enrichment in diverse settings and at-home use.

To start, patients’ experiences with cognitive screening tests suggest that undergoing screening without prior knowledge of the process or their expected performance can be strenuous and create a sense of pressure(Krohne et al., 2011), which may influence willingness to complete cognitive assessments. Older adults have also reported that cognitive screening measures can be stressful, embarrassing, and even threaten their sense of dignity (Krohne et al., 2011; Martin et al., 2015). In addition to these often-experienced negative experiences with clinician-administered cognitive screening, the recommendations provided by the USPSTF do not remedy the situation. That is, the US Preventive Services Task Force (USPSTF (Moyer & U.S. Preventive Services Task Force, 2014; Patnode et al., 2020),) recommendation statement suggests that cognitive screening for cognitive impairment does not provide any additional benefit to improve, or harm, patient and caregiver outcomes. The implications of these recommendation may create additional challenges for primary care physicians given the limited time allotted for screening patients, and limited training/resources provided for cognitive screening during Medicare Annual Wellness Visits (Jacobson et al., 2020; Petersen and Yaffe, 2020). Consequently, older adults’ willingness to engage in cognitive screening may remain limited, highlighting the need to consider acceptability and engagement when implementing cognitive measures in real-world settings Reducing these negative perspectives is therefore critical to improving feasibility and use of cognitive screening tools for dementia.

In contrast to these challenges, recent studies have investigated self-administered cognitive assessments as remote alternatives. For example, Contemori et al. (2024) used a web-based, self-administered tool (Cognitive-Cognitive Dual-Tasking; CCDT) to examine age-related differences in memory performance across cognitive-motor dual-task conditions. Their study highlights the potential of web-based cognitive testing for remote interventions. Similarly, Pucci et al. (2024) used a web-based, self-administered cognitive screening measure (Auto-Global Examination of Mental State; Auto-GEMS) to assess cognitive state, demonstrating feasibility for remote assessment, sensitivity to individual differences, accessibility, and potential for longitudinal monitoring. These advancements align with our adaptation of the Mnemonic Similarity Task (MST; S. M. Stark et al., 2019) into the optimized MST (C. E. L. Stark et al., 2023), a self-administered, open-access tool designed to assess hippocampal function and cognitive decline.

Previously, we adapted the Mnemonic Similarity Task (MST; S. M. Stark et al., 2019), a research tool sensitive to hippocampal function and cognitive decline, into the optimized MST (oMST; for details see C. E. L. Stark et al., 2023). The MST is a modified object-recognition memory paradigm that includes highly similar lures and the lure discrimination index (LDI) derived from the task has been found to tax pattern separation and is sensitive to hippocampal function (S. M. Stark et al., 2019). Blood-oxygen-level-dependent (BOLD) functional magnetic resonance imaging (fMRI) activity (Bakker et al., 2008; Kirwan and Stark, 2007; Lacy et al., 2011) and electrocorticography (ECoG (Lohnas et al., 2018);) in healthy adults have shown that subfield-specific activity in the hippocampus can discriminate between highly similar lures and repetitions using the MST. In addition, the MST has demonstrated strong test-retest reliability and is resistant to practice effects (C. E. L. Stark et al., 2023). In fact, cognitive modeling demonstrated that the MST can detect subtle changes in cognitive functioning and identify Alzheimer’s Disease biomarker status before cognitive decline becomes apparent (Vanderlip et al., 2024) making the MST an optimal tool for screening and monitoring cognition in older adults.

**Fig. 1.**
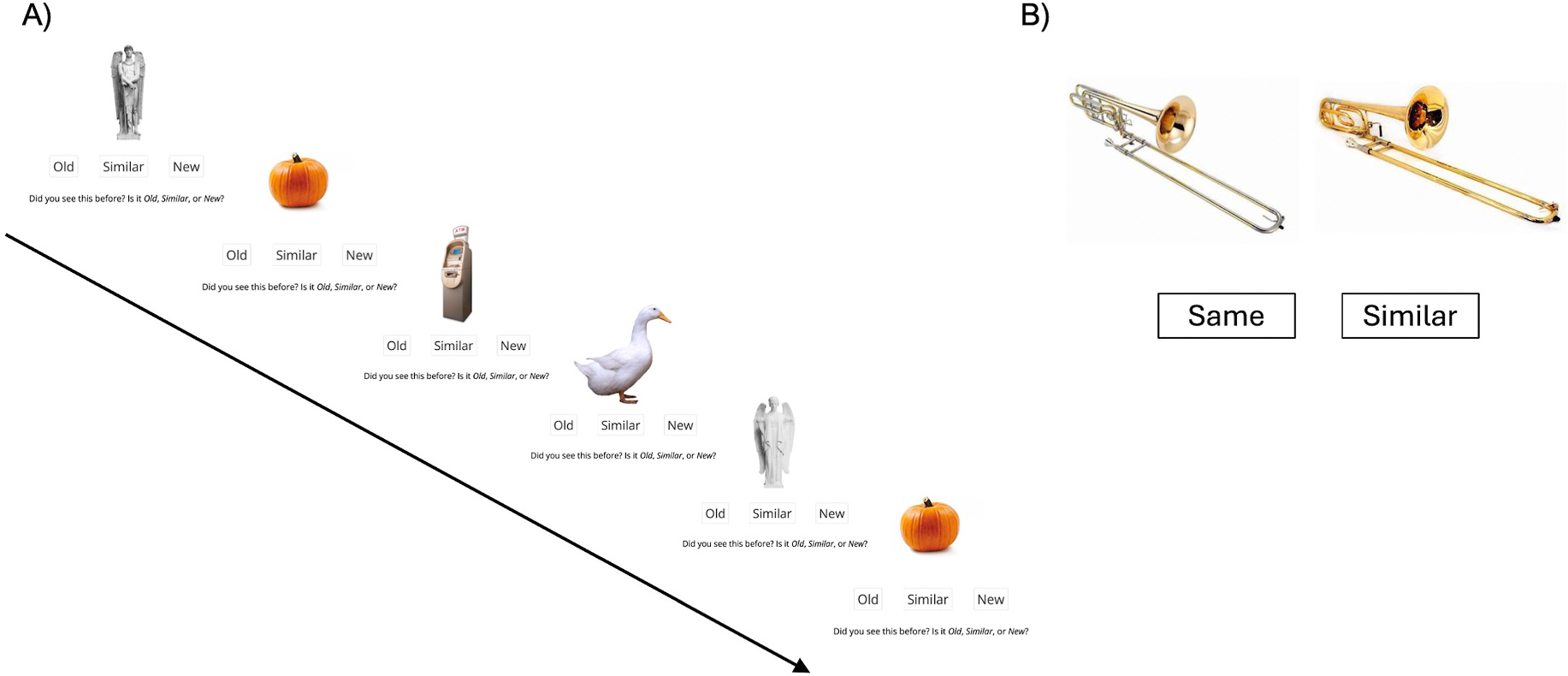
(A) Sample trials of the Optimized Mnemonic Similarity Task (oMST). Correct responses are as follows: New – New – New – New – Similar – Old. (B) Immediate Control Task (ICon) ‘*Similar’* response sample trial.

The oMST (C. E. L. Stark et al., 2023) is an automated, self-administered, and scored measure optimized for cognitive screening and population enrichment in clinical settings and at-home use, offering a potentially more neurobiologically-specific and easy to administer alternative to traditional neuropsychological tests. The oMST is a continuous recognition memory task in which participants see a series of images one at a time and classify each item as ‘Old,’ ‘Similar,’ or ‘New’ (Fig. 1A; see Methods for details). The two main outcome measures derived from this task are the recognition (REC) measure and the lure discrimination index (LDI). The REC is calculated as the probability of responding ‘Old’ to repeated items minus the probability of responding ‘Old’ to novel items. The LDI, which is the most relevant and critical outcome measure of the oMST, reflects the ability to distinguish between similar lures from old repetitions. The LDI is calculated as the probability of making a ‘similar’ response to similar lures minus the probability of identifying a novel foil as similar. The oMST is designed to accurately reflect performance similar to the research-grade MST, but in approximately 5 min. Therefore, the oMST can address the barriers observed in cognitive screening measures previously observed. In fact, older adults’ perspective on cognitive screening measures are often improved when they are given the choice to complete the screening using their preferred testing modality and testing location (Wong and Jacova, 2018). That is, many participants expressed a preference for completing a cognitive screening measure independently using either a mobile devise or computer at home.

The present study tested the reliability, validity, and accessibility of the oMST in ~1500 individuals across five experiments and eight different testing contexts. Our central questions across the five primary experiments were: 1) whether at-home, on-line testing produced similar results as lab-based, in-person testing; 2) whether testing could be reliably performed in community outreach settings that might be used for recruitment or screening; 3) whether the oMST could be reliably used in large-scale, phone-based assessments; 4) whether it could be used in the clinical research setting of an ADRC yearly evaluation; and 5) whether variations in visual acuity, often found in the older general population, would significantly impede the use of the oMST. In each experiment, we assessed the validity of the oMST in that context by comparing the resulting scores to the more controlled, in-person lab-based measures collected in Experiment 1. Importantly, the focus of the present study is on assessing reliability, validity, and accessibility to answer these research questions, rather than generating normative data for the oMST.

Unsurprisingly, we found a slight drop in performance in a number of the testing conditions that could have arisen from a wide range of factors (different overall participant populations, different selection biases, distraction, participant effort, etc.). We conducted a final set of analyses to determine whether the measures were still reflective of the underlying memory mechanisms at hand. To do this, we compared the well-documented effect of age on the MST’s LDI measure (Huffman and Stark, 2017; S. M. Stark et al., 2013, 2015; S. M. Stark and Stark, 2017; Toner et al., 2009) across contexts. We hypothesized that while there may be subtle differences in LDI overall across Experiments 1–5, if valid, the oMST’s LDI should reveal a similar age-related slope across contexts. If true, this would bolster the accessibility, reliability, and validity of the oMST as a cognitive screening tool in the wild (i.e., anywhere, anytime). The experiments reported in this article were not formally preregistered. The data will be made available on Dryad on acceptance. Requests for the data can be sent via email to the corresponding author. The oMST is freely available at: https://github.com/celstark/oMST.

## 2. Methods

A total of eight different testing contexts were used in the present work: 1) the University of California, Irvine in-person and 2) online testing; 3) the Alzheimer’s Disease Research Center (ADRC); 4) the Long Beach, CA and 5) Ontario, CA Golden Future 50+ Senior Expos; 6) the Somang Society Korean Cultural Center; 7) the Understanding America Study national online testing platform; and 8) the Prolific global online testing platform. The UCI in-person and online testing and the Prolific included participants 18+ years-old whereas the remaining testing sites included participants 40+ years-old. Participants across all testing sites completed the oMST on a computer, tablet, or mobile device. The testing modality, context, and total sample size included in the analyses for each experiment is Supplementary Table 1. As a guide for recruitment across the eight testing contexts, a priori power analyses were conducted (Faul et al., 2009). For a between-subjects *t*-test, a total sample size of 102 participants (51 per group) would provide 80 % statistical power. For a within-subjects *t*-test, 27 participants would be sufficient to achieve the same power, and for a correlation analysis, 64 participants would be needed. For the repeated-measures ANOVA comparing the two conditions (glasses vs. no glasses) across the six main tasks with age as a between-subjects factor, power to detect the within-subjects main effect is similar to that of a within-subjects *t*-test (27 participants). Detecting between-subjects effects or interactions would require larger samples depending on the expected effect sizes and group sizes; however, sample sizes in the present study were sufficient to detect medium effects with 80 % power, and the repeated-measures design reduces error variance for within-subjects comparisons. Ethnic/race breakdown is reported in each experiment and in Table 2 to illustrate the diversity of the sample and the feasibility of administering the oMST across varied participants and community contexts. No analyses of ethnic differences were conducted.

To maximize standardization across controlled laboratory and uncontrolled community or remote environments, we implemented standardized procedures across all contexts. All participants were given a self-guided instructional phase and practice trials prior to beginning the task. In the community-based events, participants were additionally offered optional earplugs to reduce distractions. While we could not control the physical testing environment during remote or community-based testing, the goal of the present work was to explicitly evaluate whether the oMST yields valid performance metrics under uncontrolled conditions, therefore assessing its feasibility and reliability across different testing contexts.

Briefly, the oMST (C. E. L. Stark et al., 2023) consists of a continuous recognition memory task in which, on each trial, participants saw sequentially presented images and were asked to make an ‘*Old*’, ‘*Similar*’, or ‘*New’* response on the currently viewed image (Fig. 1A). Old responses indicate whether the presented item is an exact repetition of a previously presented image, similar responses indicate whether the image is similar to a previously seen item but not exactly the same, and new responses indicate whether the item is an entirely new image. There are a total of 128 trials, with 64 being novel foils (first presentations of items repeated in some form subsequently), 20 being exact repetitions, and 44 being similar lure items (Fig. 1A). Lure versions have the same name as the studied items but can differ from the study item in a wide range of dimensions to mitigate the use of any particular strategy to improve performance. The number of intervening trials between an initial presentation and its repetition or lure presentation ranged from 4 to 30.

Each item appeared on screen for 2s along with a prompt instructing participants to make an ‘*Old*’, ‘*Similar*’, or ‘*New’* response using a touchscreen or by clicking on a computer mouse. After the 2s, the item disappears but the buttons remain to enable participants to make self-paced responses while fixing the available encoding duration. Lure items are divided into 5 levels of difficulty, based on prior work assessing, for each study-test pair, the probability of incorrectly judging the lure version as having been studied. In the oMST, an emphasis is made on the more dissimilar items (5, 6, 11, 11, and 11 items from each of the 5 lure bins). Between this, and the shorter interval between first and second exposures to an item relative to the classic study-test MST, overall performance is elevated to eliminate floor effects in older or mildly impaired populations. Stimuli were displayed on a white background within a 400 × 400-pixel grid.

Prior to the experimental session, participants received a self-guided instructional phase with two practice trials highlighting examples of an ‘*Old’* response trial and a ‘*Similar*’ response trial. Initial trials indicate explicitly what subjects should respond, while later trials are undirected, but provide feedback and force an eventual correct response. These are here to ensure that participants understand what we mean by ‘*Old’* vs. ‘*Similar’*. All participants across testing contexts completed this same guided instruction and practice phase, ensuring that understanding of the task demands was standardized regardless of setting. All tasks were implemented using the open-source jsPsych library for web-based deployment (de Leeuw, 2015) and the open-source JATOS package (Lange et al., 2015) to provide a reliable means of securely administering test sessions on the web and managing the data.

In all experiments, there were two primary measures can be derived from responses made on the oMST. First, a recognition (REC) measure is calculated using probability of responding ‘old’ to items previously seen minus the probability of responding ‘old’ to novel items. This is the “corrected recognition memory” score often used in the literature to account for subject response biases. Consistent with our prior work, participants with REC scores under 0.5 were excluded from further analyses (their data are plotted, however), as scores below this threshold likely indicate unreliable responses. Below-threshold recognition memory performance could be due to several reasons, including misunderstanding of the task instructions, lack of attention or desire to engage in the task, potentially clinically-relevant memory impairments, and/or severe bias in use of particular button responses. Therefore, these participants were judged as not able to complete the experiment as instructed and were removed from all analyses. In the Stark et al. (2023) optimizing the MST, 20.73 % of young adult data and 12.28 % of older adult data were rejected due to invalid REC scores using similar online testing. Note that here, our overall exclusion rate was 8.59 % (analyses using all data are present in the Supplementary Materials).

The second, and most-relevant measure, is the lure discrimination index (LDI), designed to evaluate participant’s ability to discriminate between lures as being similar unique items rather than an old repetition of a previously viewed item. It is derived using the probability of making a ‘similar’ response to similar lures minus the probability of identifying a novel foil as similar. Like the REC, this difference score is designed to adjust for response biases. Although participants were not excluded for having low LDI scores, as they were for below-threshold REC scores, we examined the proportion of individuals with LDI scores below 0.10 as an outcome measure to compare with Stark et al. (2023). In that study, 18.29 % of young adults and 23.21 % of older adults had LDI scores below the arbitrary threshold of 0.10, suggesting an extremely poor ability to discriminate between similar lures and targets.

A Shapiro-Wilk test was conducted to assess normality for both REC and LDI prior to statistical analyses. If the data met normality assumptions, parametric tests were used; otherwise, non-parametric tests were used. REC and LDI data analysis procedures are identical for all experiments.

The oMST is freely available on GitHub in several formats. In prior work, Stark et al. (2023) employed a web-based and open-source JATOS package to reliably administer the task on the web and manage data. Experiments described in this study utilized a similar structure.

## 3. Results

### 3.1. Experiment 1

Experiment 1 aimed to assess the relationship between in-person researcher-administered and remote self-administration of the oMST using a within-subject design in both younger and older adults.

#### 3.1.1. Participants

Experiment 1 recruited a total of 83 participants over the age of 18-years-old to ensure sufficient representation across the age ranges. Data from six participants were excluded from statistical analyses due recognition memory performance below the 0.5 REC threshold (n = 3) and/or invalid data. Therefore, 77 participants were included in the analyses for Experiment 1. The sample consisted of 31 young adults (72.41 % Female; *M*_age_ = 22.52, *SD*_age_ = 4.80; 21.43 % White/Non-Hispanic, 57.14 % Asian, 17.86 % Hispanic, 3.57 % Black/African American) and 46 middle aged and older adults (64.58 % Female; *M*_age_ = 69.48, *SD*_age_ = 12.50; 89.58 % White/Non-Hispanic, 6.25 % Asian, 4.17 % Hispanic) from Irvine, CA and surrounding cities. Participants recruited from UC Irvine were enrolled in introductory psychology courses and received course credit for their participation. Participants recruited from the city of Irvine, CA and surrounding communities were enrolled in UCI’s Consent to Contact database and compensated $15/hr for their participation. Our inclusion criteria for required that all participants recruited from UCI’s Consent to Contact database live in Orange County, speak English, and be 18+ years old. Participants who reported REM sleep disorders, visual or hearing impairments (i.e., blindness or deafness), suffered from head trauma with loss of consciousness, diagnosed with cancer, and/or neurological disorders were excluded from the study.

All research procedures in the present study across the 6 Experiments were approved by the Institutional Review Board of University of California, Irvine (IRB HS #2008–6128). All participants were provided an informed consent and were compensated for participation by course credit (undergraduate students) or monetary compensation (community participants).

#### 3.1.2. Procedure

Participants completed the optimized Mnemonic Similarity Task (oMST (C. E. L. Stark et al., 2023); across two different test administration settings. In one condition, participants completed the oMST on the UCI campus with a researcher present for the first session and complete the task again remotely for the second session. In the other condition, participants were asked to complete the task remotely for the first session and in person with a researcher for the second session. Conditions were counterbalanced across participants. In each condition participants received a new set of stimuli.

#### 3.1.3. Results

**Fig. 2.**
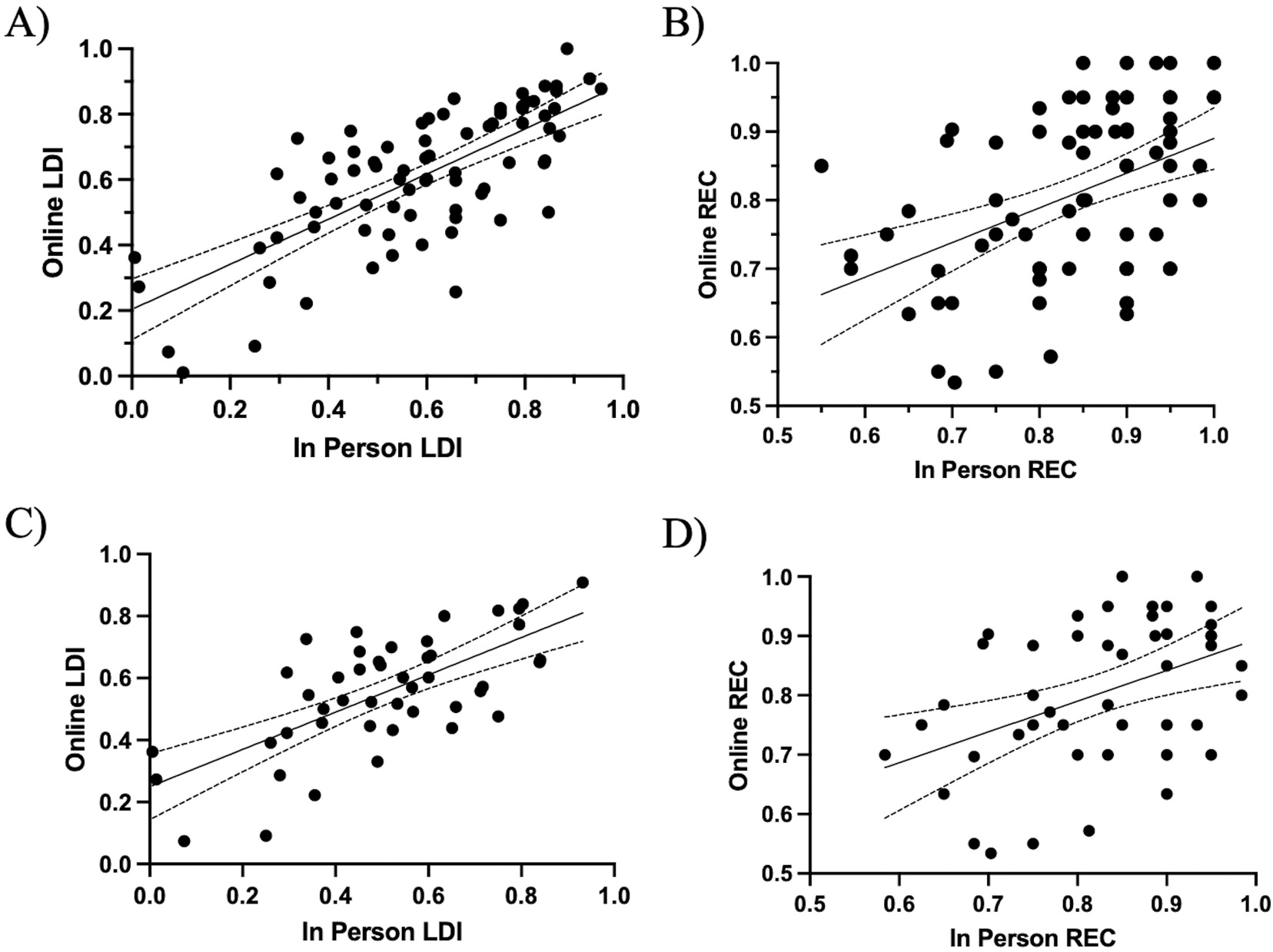
Relationship between in person and online LDI (A) and REC (B) across all ages. Relationship between LDI (C) and REC (D) for individuals 40+.

**Fig. 3.**
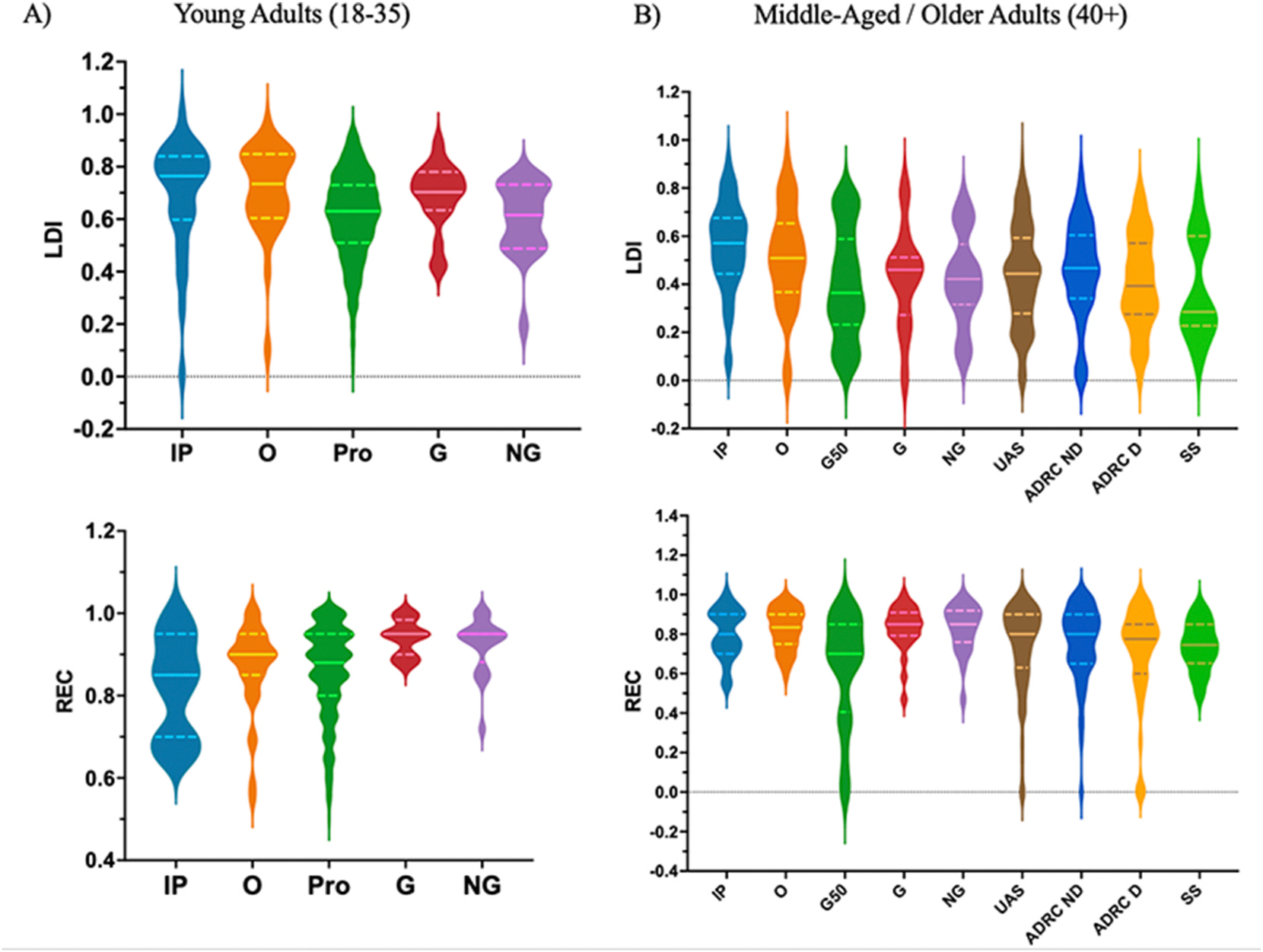
LDI and REC across testing sites and age groups (Young Adults *n* = 1051; Middle-Aged/Older Adults *n* = 634). IP: UCI In-person (n = 83); O: UCI Online (n = 83); G50: Golden Future 50+ Senior Expo (n = 60); UAS: Understanding America Study (n = 214); Pro: Prolific (n = 999); ADRC ND: UCI ADRC No Dementia group (n = 119); ADRC D: UCI ADRC Dementia group (n = 145); G: UCI Glasses; NG (n = 46): UCI No Glasses (n = 46); SS: Somang Society (n = 18). Note, all participants’ scores, including those removed from statistical comparisons for below threshold REC, are included here. (For interpretation of the references to color in this figure legend, the reader is referred to the Web version of this article.)

Of primary interest, the relationship between in person and online oMST LDI was assessed (Fig. 2A). A bivariate Pearson paired samples correlation found a statistically significant association between in person and online LDI, *r* (75) = 0.73, *p* < .001, across all participants. This suggests a strong relationship between lure discrimination performance, regardless of whether the oMST was self-administered or administered in-person by a researcher. Next, we investigated the relationship between REC for in person and online oMST administration across all age groups (Fig. 2B). As REC scores in young adults in particular were impacted by ceiling effects (Fig. 3), a Spearman correlation was used and revealed a reliable correlation between in person researcher administered and online self-administered scores (r (75) = .43, *p* < .001). The significant correlations between in-person and remote LDI and REC scores further suggest strong cross-context reliability of the oMST across both modes of test administration.

As a confirmatory analysis, the relationship between in person and online LDI and REC scores were analyzed for participants 40+ years old to ensure that the observed effects were not skewed by data from younger adults (Fig. 2C & D). Similar to the whole-group analysis, there was a reliable association between in person researcher-administered and online self-administered LDI (*r* = 0.69, *p* < .001) REC (*r* = 0.44, *p* = .002) in those over 40. These results suggest that regardless of testing location, administration mode, and age the oMST can reliability capture recognition memory performance and lure discrimination.

Lastly, we ran two separate repeated measures ANOVAs to test whether order effects (i.e., completing the task within person first vs. online first) influenced oMST performance. There was no statistically reliable effect of order for the In Person-first vs. Online-first in either younger (LDI F (1,28) = 0.04, p = .842; REC F (1,28) = 3.78, p = .062) or middle aged and older (LDI F (1,45) = 2.71, p = .107; REC F (1,45) = 2.49, p = .122) adults. These results indicate that the observed effects were not heavily impacted by practice effects.

### 3.2. Experiment 2

Experiment 1 demonstrated that the oMST reliably measures recognition memory and lure discrimination when administered in person by a researcher or remotely self-administered. To further assess the validity of the oMST as a cognitive screening tool in community settings, Experiment 2 explored its accessibility and applicability across diverse communities and testing environments. Data for Experiment 2 were collected onsite at two Golden Future 50+ Senior Expos held in Ontario, CA, and Long Beach, CA and the Somang Society Korean Cultural Center in Cypress, CA.

#### 3.2.1. Participants

Data from 58 participants were collected from the Golden Future 50+ Senior Expos and from 18 participants was collected from the Somang Society Korean Cultural Center. However, given the nature of onsite data collection at the expos (i.e., real-time visual and auditory distractions) which could have contributed to REC scores below the .50 threshold, Experiment 2 only included data from 44 participants from the Golden Future 50+ Senior Expo (77.27 % Female; *M*_age_ = 65.00, *SD*_age_ = 9.05; 31.82 % Asian, 25 % Hispanic, 31.82 % White/Non-Hispanic, 2.27 % Black/African American, 9.09 % More than one race) and 14 participants from the Somang Society Korean Cultural Center (64.29 % Female,; *M*_age_ = 70.00, *SD*_age_ = 8.12; 92.82 % Asian, 7.14 % White/Non-Hispanic). Data from 14 participants from the Golden Future 50+ Senior Expos and 4 participants from the Somang Society were excluded from the study due recognition memory performance below the 0.5 REC threshold and/or invalid data. Our inclusion criteria required that all participants be 50 years or older and possess sufficient English proficiency to read and understand the task instructions and complete the self-administered task.

#### 3.2.2. Procedure

Stimuli and task procedures were identical to Experiment 1. An exhibitor booth was setup in the Golden Future 50+ Senior Expos. Flyer and banner advertisements for the study were present on and near the testing booth. Interested participants were provided with a brief description of the study and were asked to complete the study task on a touch screen laptop provided by the experimenters. Participants did not receive monetary compensation for completing the task; however, they were given a rubber duck toy as a token of appreciation for their time (the task was not only called “the rubber duckie task” for many years, but these ducks and their variety demonstrate quite clearly what we mean by “similar”).

Similarly, interested participants at the Somang Society Korean Cultural Center completed the task on-site during the cultural center’s scheduled social event. The cultural center staff were provided recruitment flyers and touch screen laptops for participants to self-administer the oMST. The Somang Society was provided with compensation of $300 for staff and participant compensation.

#### 3.2.3. Results

First, two separate analyses were run to test the mean difference in REC and LDI scores between the two Golden Future 50+ Senior Expos, and the Somang Society Korean Cultural Center. There was no statistically reliable mean difference for LDI between the Ontario, CA (*M* = .37, *SD* = .29), the Long Beach, CA (*M* = .39, *SD* = .20) expo data, and the Somang Society (*M* = .38, *SD* = .22; *F* (2, 55) = 0.02, *p* = .985). Similarly, a Kruskal-Wallis test indicated that there was no significant difference in REC across the Ontario, CA (Mdn = .75), Long Beach, CA (Mdn = .80), and the Somang Society data (Mdn = .74; χ^2^ (3, *N* = 58) = 1.66, *p* = .559). Therefore, data from both Golden Future 50+ Senior Expos and Somang Society were combined in subsequent analyses.

Next, of primary interest, two separate analyses were run to test the mean difference in LDI and REC scores between data collected during the community outreach events (Golden Future 50+ Senior Expos and Somang Society Korean Cultural Center) and age-matched in-person at UCI (Experiment 1; Fig. 3). We observed a statistically reliable decrease of 0.13 (0.82 sd) in LDI scores between community outreach events (*M* = .38, *SD* = .21) and in-person lab (*M* = .51, *SD* = .21) data (*t* (99) = 2.79, *p* = .006). We also observed a statistically reliable decrease of 0.05 (0.30 sd) in REC scores between the community outreach events (*M* = .77, *SD* = .12) and in-person lab (*M* = .82, *SD* = .11) data (*t* (99) = 2.70, *p* = .011). One possibility for these significant differences in mean LDI and REC could be that participants who completed the task at the community events were faced with more visual and auditory distractors from their surrounding than those who completed the task in a quite private testing room at the university. In fact, according to the Inhibitory Deficit Hypothesis (Hasher and Zacks, 1988) older adults may experience reduced inhibitory regulation (i.e., task-irrelevant distractors during the community events) and may experience heightened distractibility, leading to reduced recognition memory performance and worse lure discrimination. It is certainly also possible that we are sampling qualitatively different populations along a host of relevant dimensions that might impact memory performance. Despite these potential issues, both LDI and REC scores remained well-distributed, albeit somewhat lower.

### 3.3. Experiment 3

Experiments 1 and 2 demonstrated that although there are some modest reductions in LDI and REC across in-person testing with a researcher and testing in a community setting where distractors are present, the oMST yielded robust results (see Experiment 6 for details). In addition, Experiment 1 demonstrated a strong correlation between in-person and remote testing. Here, we aimed to test the reliability of oMST outcome measures on national and global online testing platforms with the goal of validating the oMST as a cognitive screening measure that can be reliably used anywhere.

#### 3.3.1. Participants

Two hundred and fourteen participants were recruited from the Understanding America Study (UAS) online testing platform, which is available to United States residents (56.82 % Female; *M*_age_ = 64.51, *SD*_age_ = 12.84; 6.36 % Asian, 3.64 % Hispanic, 77.73 % White/Non-Hispanic, 12.27 % Black/African American). The UAS is maintained by the Center for Economic and Social Research (CESR) at the University of Southern California, which oversees survey administration and participant compensation. Participants enrolled in the UAS receive $20 for every 30 min spent completing surveys on the testing platform. Following the UAS inclusion criteria, the present study required that participants reside in the United States, have sufficient English proficiency to read and understand the task instructions and complete the self-administered task. In addition, participants were required to be 40 years old or older. Of the 214 recruited participants, 28 were excluded based on recognition memory performance below the 0.50 REC threshold and/or invalid data, resulting in a final sample of 186 participants included in the analyses.

In addition, data from 999 participants was collected from the Prolific online global testing platform (50.86 % Female; *M*_age_ = 26.23, *SD*_age_ = 4.29; 6.08 % Asian, 32.81 % Black, 51.34 % White/Non-Hispanic, 0.40 % Native American, 9.37 % More than one race). Participants recruited from Prolific reported residing in 23 different countries (5 continents; Table 2). Participants recruited from Prolific are compensated at an hourly rate of $8.00, prorated based on the time spent completing a survey. Of the 999 recruited participants, 62 were excluded based on recognition memory performance below the 0.50 REC threshold and/or invalid data, resulting in a final sample of 937 participants included in the analyses. Inclusion criteria for Prolific required that participants be young adults (18–35 years old) and possess sufficient English proficiency to read and understand the task instructions and complete the self-administered task.

#### 3.3.2. Procedures

oMST stimuli and task procedures were identical to the prior experiments. Being fully-remote, however, participants completed the task on their own device in settings of their choosing. For the UAS, the code was re-implemented into their testing platform. For Prolific, the same jsPsych/JATOS testing platform from the prior experiments was used.

#### 3.3.3. Results

Given the highly diverse sample in Experiment 3, we first aimed to examine mean differences in performance on the two oMST outcome measures (LDI and REC) across the two online testing platforms. Next, we investigated mean differences in LDI and REC across UAS, Prolific, and age-matched data from UCI (Fig. 3). We compared these with both in-person and online data from Experiment 1, given the strong correlation observed between in-person and online LDI and REC, as well as the online nature of data collection in Experiment 3.

First, we investigated the mean difference in LDI and REC between age-matched data from Experiment 1 and data obtained from UAS. A *t*-test indicated a significant difference in LDI (*t* (230) = 3.60, *p* < .001) across UAS (*M* = .44, *SD* = .20) and UCI online age-matched data (*M* = .56, *SD* = .19). However, a Mann-Whitney test indicated that there was no significant difference in REC across the UAS (Mdn = .83, *M* = .80, *SD* = .13) and UCI online age-matched data (Mdn = .80, *M* = .80, *SD* = .13; *U* = 4181, *p* = .812).

Next, we investigated the mean differences in LDI and REC between age-matched data from Experiment 1 and data obtained from Prolific. Similar to the results observed with the UAS and UCI age-matched data, a *t*-test indicated a significant difference in LDI across the Prolific online testing platform (*M* = .61, *SD* = .17) and UCI online (*M* = .67, *SD* = .23) age-matched data, *t* (968) = 2.10, *p* = .036. Additionally, a Mann-Whitney test indicated that there was a significant difference in REC across the Prolific online testing platform (Mdn = .89, *M* = .86, *SD* = .11) and UCI online age-matched data (Mdn = .85, *M* = .78, *SD* = .19; *U* = 11,854, *p* = .021). It is worth noting that as in Experiment 1 and prior work, REC, and to some degree LDI scores in young, healthy adults suffer from ceiling effects and may necessitate non-parametric statistical measures.

In addition to the oMST, we aimed to better understand the perspective of participants and willingness to participate again in the oMST as a cognitive screening measure. Participants in the UAS were asked to rate the amount of effort they used to remember each image and accurately respond throughout the beginning and end of the task, with responses ranging from 1 (‘*little effort*’) to 3 (‘*maximal effort*’). They were also asked to rate how likely they are to participate in the oMST as a cognitive screenings assessment in the future, rated on a scale from 1 (‘*definitely not*’) to 5 (‘*definitely will’*). Lastly, participants were asked to rate how interesting they found the task, with 1 representing ‘*very uninteresting*’ and 5 representing ‘*very interesting*’. A paired-samples *t*-test comparing the mean difference in effort exerted to accurately remember the images and complete the task revealed that participants exerted significantly more effort toward the end of the task (*M* = 2.41, *SD* = 0.66) than the beginning of the task (*M* = 2.19, *SD* = 0.68; *t* (213) = 4.50, *p* < .001). While participants self-reported exerting significantly more effort toward the end of the task, the majority of participants found the task either ‘*very interesting*’ or ‘*interesting’* (79 %). In addition, 68 % of the participants reported that they will ‘*definitely’* or ‘*probably’* be willing to complete this online cognitive screening assessment again in the future.

### 3.4. Experiment 4

Experiments 1 and 2 demonstrated that the oMST can yield reliable results regardless of task completion with a researcher, remotely, or in the uncontrolled settings of a community event. Next, we tested the feasibility and reliability of the oMST in a clinical research environment, testing participants in UCI’s Alzheimer’s Disease Research Center (ADRC). Following the UCI ADRC inclusion criteria, all participants were required to be 60 years or older and have sufficient English proficiency to read and understand the task instructions and complete the self-administered task.

#### 3.4.1. Participants and procedures

265 participants from UCI’s ADRC were recruited as part of their annual visits to complete the oMST (60.75 % Female, 39.25 % Male; *M*_age_ = 75.74, *SD*_age_ = 6.66; 19.31 % Asian, 7.72 % Hispanic, 66.80 % White/Non-Hispanic, 5.79 % American Indian, 0.39 % More than one race). oMST stimuli and procedures were identical to Experiment 1. Participants from the ADRC completed a larger testing battery, which included the oMST and the Dementia Severity Rating Scale (DSRS). The DSRS is an 11-item multiple-choice questionnaire assessing functional abilities, which include memory, personal care, mobility, etc. The total score ranges from 0 to 36 where higher scores indicate more dementia severity. Using the DSRS total score a clinical dementia rating score (CDR) was calculated ranging from 0 to 2. Participants were categorized as follows: scores of 0–2 on the DSRS were coded as 0 (No Dementia, *n* = 119), scores of 2–11 as 0.5 (Questionable Dementia, *n* = 135), scores of 12–22 as 1 (Mild Cognitive Impairment, MCI, *n* = 10), and scores of 23–36 as 2 (Moderate Cognitive Impairment, *n* = 1).

#### 3.4.2. Results

We began by assessing the oMST performance in ADRC participants who scored 0 (No Dementia) on the CDR, comparing them to age-matched participants from Experiment 1. We found that there was no significant mean difference in LDI between the in-person data from Experiment 1 (*M* = .49, *SD* = .21) and the unimpaired ADRC cohort (*M* = .45, *SD* = .20), *t* (148) = 1.63, *p* = .247 (Fig. 3). Similarly, a Mann-Whitney *U* test indicated that there was no significant difference in REC between in-person data from Experiment 1 (Mdn = .83, *M* = .82, *SD* = .11) and this cohort (Mdn = .80, *M* = .79, *SD* = .13; *U* = 1921, *p* = .186). These results support the idea that the oMST is a viable testing platform within clinical research settings such as the nationwide ADRC network.

Next, we aimed to investigate whether the shortened oMST variant in use here would be able to identify early stages of dementia the way the classic MST has (Belliart-Guérin and Planche, 2023; S. M. Stark et al., 2013; Vanderlip et al., 2024). Here, we combined the Questionable Dementia (n = 135) and MCI (n = 10) CDR groups into an Early Impairment group as the MCI group (n = 10) was too modest to stand on its own and as there was no significant mean difference between the Questionable Dementia and MCI groups for LDI (*t* (143) = 1.12, *p* = .261) or REC (U = 472, p = .114). As expected, and consistent with previous findings, we found a significant mean difference in LDI (*t* (262) = 1.99, *p* = .048) between the No Dementia (*M* = .46, *SD* = .20) and the Early Impairment (*M* = .41, *SD* = .20) groups. However, the Mann-Whitney *U* test did not indicate a significant difference in REC (U = 7847, p = .206) between the No Dementia (Mdn = .80, *M* = .75, *SD* = .19) and the Early Impairment (Mdn = .78, *M* = .71, *SD* = .25) groups.

### 3.5. Experiment 5

Experiments 1–4 demonstrated that the oMST yields reliable results regardless of mode of task administration. However, the possibility still remains that individual differences across visual acuity, which may decline as part of normal aging, could be a contributing factor to the age effects. Since visual acuity, is associated with cognitive impairment (Chen et al., 2017; Harrabi et al., 2015) a key concern in interpreting oMST results is how well participants can perceive the objects and how well they can encode them into any form of memory. Therefore, in Experiment 5 we sought to further assess the impact of visual acuity on lure discrimination by directly manipulating it. We recruited participants who wear glasses or corrective lenses to complete the task both with and without their glasses.

#### 3.5.1. Participants

Experiment 5 recruited a total of 46 participants between the ages of 18 and 92 years old (57.89 % Female; *M*_age_ = 54.86, *SD*_age_ = 23.73; 57.89 % White/Non-Hispanic, 28.95 % Asian, 13.16 % More than one race) from Irvine, CA and surrounding communities. Participants were enrolled in UCI’s Consent to Contact database and compensated $15/hr for their participation. The UCI consent to contact database participant inclusion and exclusion criteria were identical to Experiment 1, with the additional inclusion criteria that participants must wear glasses or contacts to be eligible for this study. Additional data from four participants were excluded from data analysis due to low REC scores.

#### 3.5.2. Procedure and measures

##### oMST.

The stimuli and oMST task procedures were identical to those in Experiment 1. In addition to the oMST, participants completed three different assessments of visual acuity. In each condition (Glasses vs No Glasses) participants received a new set of stimuli.

##### Tumbling E.

First, all participants completed a computerized Tumbling E visual acuity test on the same computer used for the oMST. In this task, the letter ‘E’ was displayed on the screen in one of four orientations—upward, downward, rightward, or leftward—corresponding to 0-, 90-, 180-, and 270-degrees. For each orientation, the letter ‘E’ was displayed in sizes of 6, 7, 8, 10, 15, 20, 25, 30, and 50 pixels, with a total of 36 trials. Participants were required to determine and indicate the direction in which the letter was facing. Tumbling E scores represent percent accuracy where higher values indicate better accuracy. Notably, by being on the same device used for the oMST, the Tumbling E task captures visual acuity at the same “intermediate” distance.

##### Snellen Chart.

Next, participants completed the Snellen chart test to assess visual clarity and sharpness for traditional distance vision. They stood 20 feet away from the chart and read each line from top to bottom. If a participant was unable to correctly identify more than three letters on a given line, their Snellen score was recorded, ranging from 0.1 to 2. A score of 20/10 (or 2) represents the highest level of visual acuity, indicating the ability to see details at 20 feet away. Conversely, a score of 20/200 (or 0.1) represents the lowest level of acuity on the chart, meaning the participant can only distinguish details 1 foot away.

##### MNREAD Acuity Chart.

Participants also completed the MNREAD Acuity Chart, which evaluates both reading speed and reading acuity at close distances. For this test, participants stood 16 inches (40 cm) from the chart and read each sentence aloud from top to bottom. The smallest font size each participant could read was recorded as their MNREAD M-size score, ranging from 0.13 to 4.0. Lower M-size scores indicate better visual acuity, while higher scores reflect the need for larger print to read comfortably. Participants repeated this counterbalanced procedure with and without their glasses or contacts and using different text passages.

##### Immediate Control Task (ICon).

Participants completed a sequential and simultaneous ICon (Fig. 1B). The ICon was used to assess visual acuity, object perception, working memory, and attention. In the sequential ICon, participants viewed two sequential images for 2 s each, with an intervening mask presented for 2 s between the images. While viewing the second image, participants were prompted to make a ‘*Same’* or ‘*Similar’* response. The prompt remained on screen until a response was made. The total time to complete the sequential ICon was 3 min. In the simultaneous ICon, participants viewed two simultaneously presented images and were prompted to make a *Same’* or ‘*Similar’* response. The prompt remained on screen until a response was made. Stimuli on both ICons were displayed on a white background within a 400 × 400-pixel grid. The total time to complete the simultaneous ICon was 1.5 min.

#### 3.5.3. Results

First, we aimed to determine if our manipulation of glasses vs no glasses worked by comparing the results of the computerized Tumbling E, Snellen chart, and the MNREAD Acuity Chart. We ran three different paired samples *t*-test for each outcome measure and found a statistically significant differences between the glasses and no glasses conditions for the Tumbling E, Snellen chart, and the MNREAD Acuity Chart (Table 1; *p*’s < 0.001). These results confirm that, as expected, visual acuity improves with the use of corrective lenses for individuals who require them. Next, we aimed to further assess the validity of the manipulation by comparing the mean difference between glasses vs no glasses for both the sequential and simultaneous ICons and found that there were no remotely reliable differences between the glasses and no glasses conditions for both tasks (Table 1; *p*’s > 0.2).

**Fig. 4.**
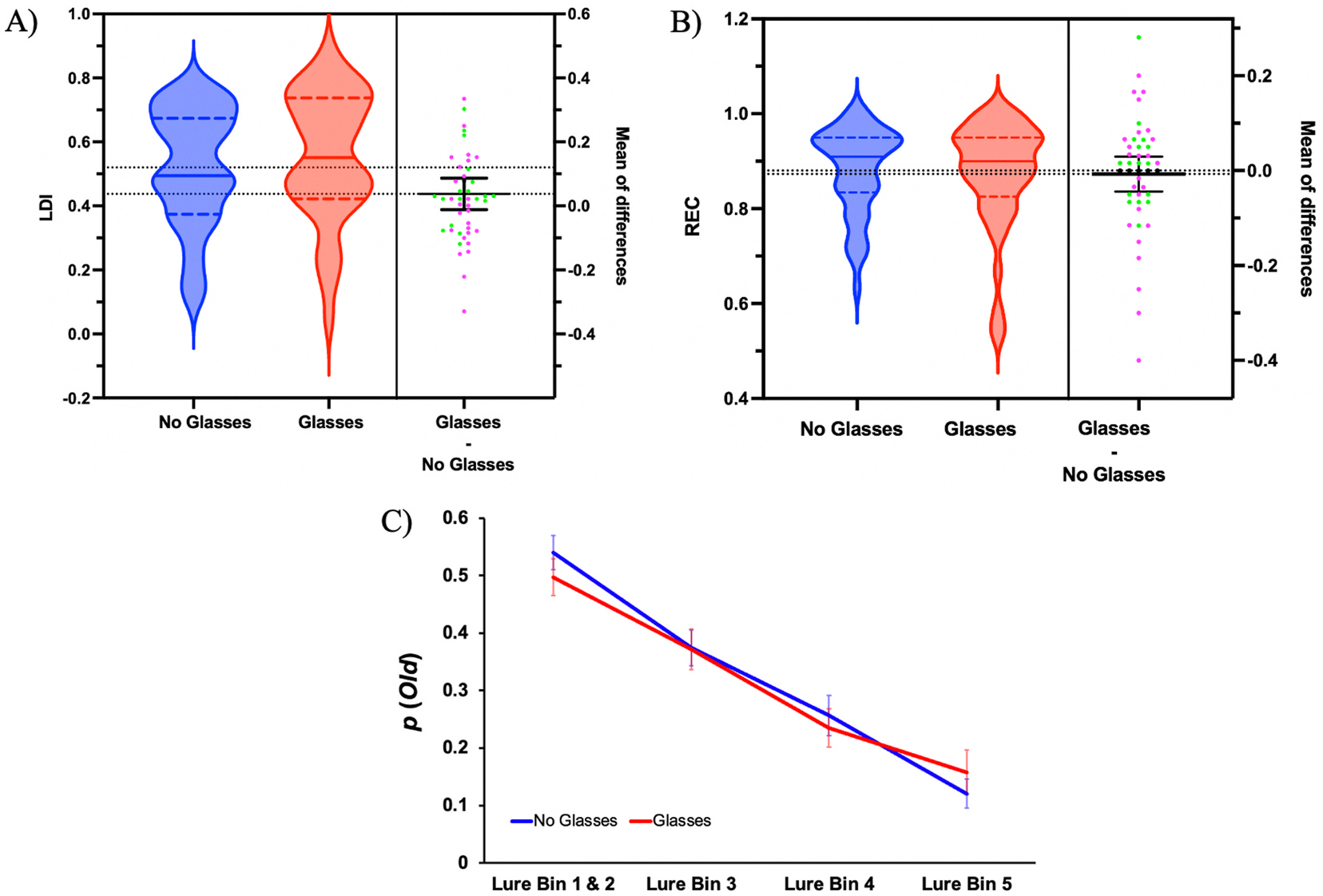
LDI (A) and REC (B) for the within-subject No Glasses and Glasses conditions. Mean of differences age groups: Younger Adults (Green) and Middle-Aged and Older Adults (Magenta). (C) The probability of responding ‘*Old’* across lure bins for the No Glasses and Glasses conditions. (For interpretation of the references to color in this figure legend, the reader is referred to the Web version of this article.)

More importantly, to examine the role of visual acuity on oMST performance, two repeated-measures ANOVAs were run, one for LDI and one for REC, with age group (young adults: 18–35; middle-aged and older adults: 40+) as the between-subjects factor, comparing the glasses and no-glasses conditions for both measures. For LDI, there was no reliable interaction between glasses condition and age group (*F* (1, 44) = 0.92, *p* = .342) and no reliable main effect of glasses condition (*F* (1, 44) = 3.28, *p* = .077) with mean LDI scores of 0.56 (SD = 0.20) for the glasses condition and 0.52 (SD = 0.18) for the no-glasses condition (Fig. 4A). Similarly, for REC, there was no reliable interaction (*F* (1, 44) = 0.92, *p* = .342) and no main effect of glasses condition (*F* (1, 44) = 0.05, *p* = .828) with mean REC scores of 0.87 (SD = 0.11) for the glasses condition and 0.88 (SD = 0.09) for the no-glasses condition (Fig. 4B). These findings indicate that the age effects observed in Experiment 1 on lure discrimination were not attributable to age-related declines in visual acuity.

Next, we ran two separate repeated measures ANOVAs to test whether order effects (i.e., completing the task with glasses first vs. without glasses first) influenced oMST performance. There was no statistically significant effect of order for the Glasses vs. No Glasses Experiment on LDI (*F* (1,45) = 0.03, *p* = .132) or REC (*F* (1,45) = 0.15, *p* = .697). These results indicate that the observed effects were not driven by practice effects.

Lastly, a repeated measures 2 × 2 ANOVA was conducted to examine mean differences in the probability of responding *‘Old’* across the different lure bins and between the Glasses and No Glasses conditions (Fig. 4C). Lure bins 1 and 2 were combined into a single group, consisting of 11 stimuli, to match the size in lure bins 3–5. There was no reliable difference in the probability of responding *‘Old’* between the Glasses and No Glasses conditions across the different lure bins (*F* (1,3) = 1.04, *p* = .381, *η*^*2*^ = .01). These further results suggest that despite degrading in visual acuity in the No Glasses condition, participants were still able to perceive the stimuli and respond at comparable rates across the lure bins for the probability of identifying an item as *‘Old’*.

### 3.6. Cross-experiment validation

Across Experiments 1–5 we observed subtle but reliable differences in LDI and REC scores across the diverse testing locations. Here, we sought to test whether, despite these overall modest shifts, the oMST was reflecting similar aspects of memory function and whether it truly was a viable test in these contexts. We did this by addressing two key questions: 1) whether the age effects in LDI (and REC) were consistent across experiments and 2) whether the rate of test failures was comparable. For these purposes, we excluded data from the UCI ADRC Dementia group due to the significant difference observed between the UCI ADRC Dementia and No Dementia groups (see Experiment 4), which aligns with previous findings (Belliart-Guérin and Planche, 2023; Vanderlip et al., 2024). Additionally, data from the Somang Society group (Experiment 2) were excluded given the small sample size (N = 14) and restricted age range.

#### 3.6.1. Results

##### Age-Effects and LDI/REC.

Eight separate correlations for LDI and REC for the eight testing sites across the 5 different experiments were run in order to observe age effects, if any, are present (Table 3). It is important to note that age effects across independent testing sites may not be present if the variability across age is limited. We observed a reliable correlation between age and LDI across all testing sites except the Golden Future 50+ Senior Expo and the Prolific data sets (see Table 3). In addition, we observed a reliable correlation between age and REC for the UCI Online (*r* (75) = −. 24, *p* = .036), UCI Glasses (*r* (44) = −. 49, *p* < .001), and UCI No Glasses (*r* (44) = −. 51, *p* < .001), and UAS (*r* (184) = −. 16, *p* = .031) data sets.

**Fig. 5.**
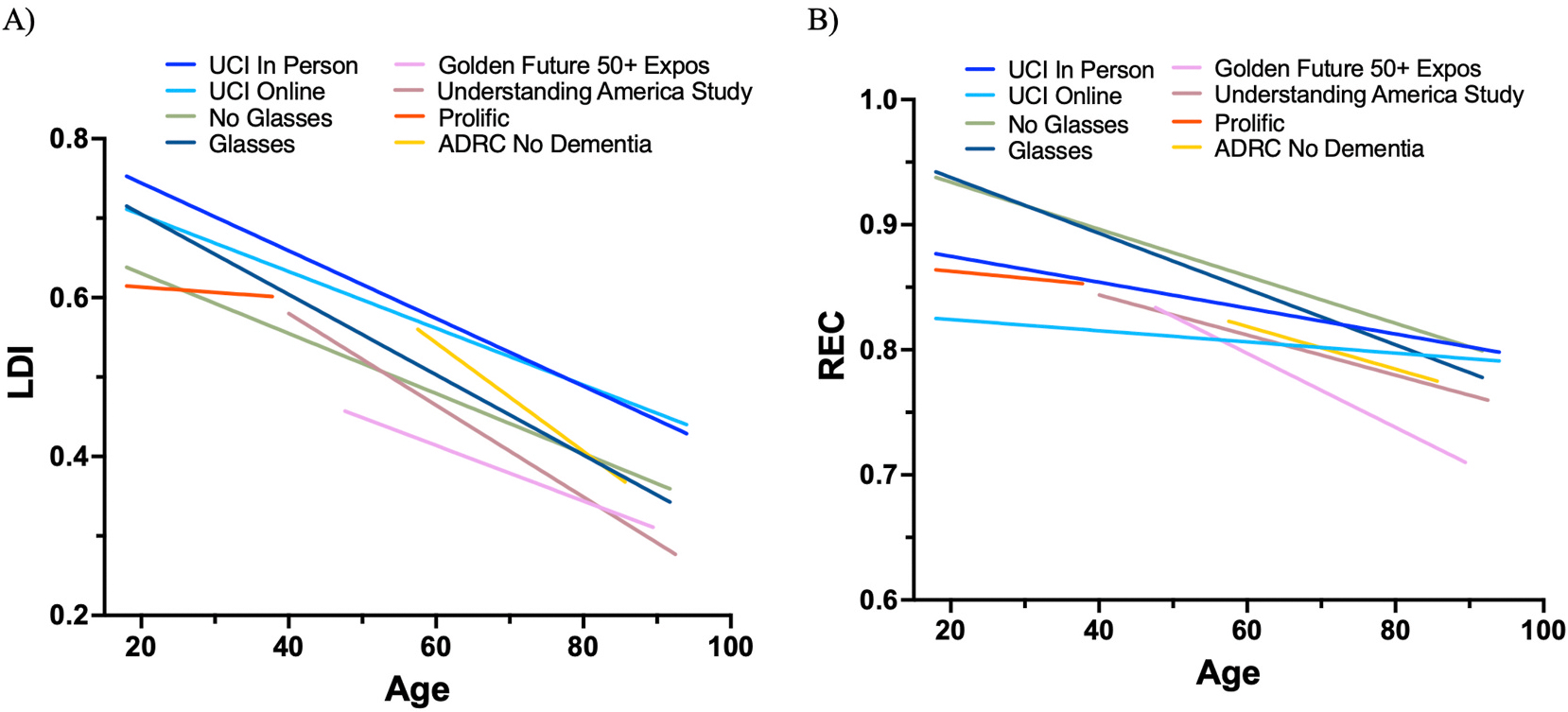
Regression analyses examining age-effects across (A) LDI and (B) REC for the different testing contexts.

Next, and more importantly, the slopes investigating age effects observed in REC and LDI across the different testing sites were compared to determine if there was a significant difference between the slopes (Fig. 5). Two separate regression analyses were run and revealed that there was no difference between the slopes across testing contexts for LDI (*F* (7, 1505) = 1.66, *p* = .114) and REC (*F* (7, 1505) = 1.04, *p* = .403). These results suggest that the age and LDI/REC did not significantly differ between the testing sites further demonstrating the validity and reliability of the oMST as a cognitive screening tool in diverse settings and populations.

##### Rate of Invalid REC and LDI.

**Fig. 6.**
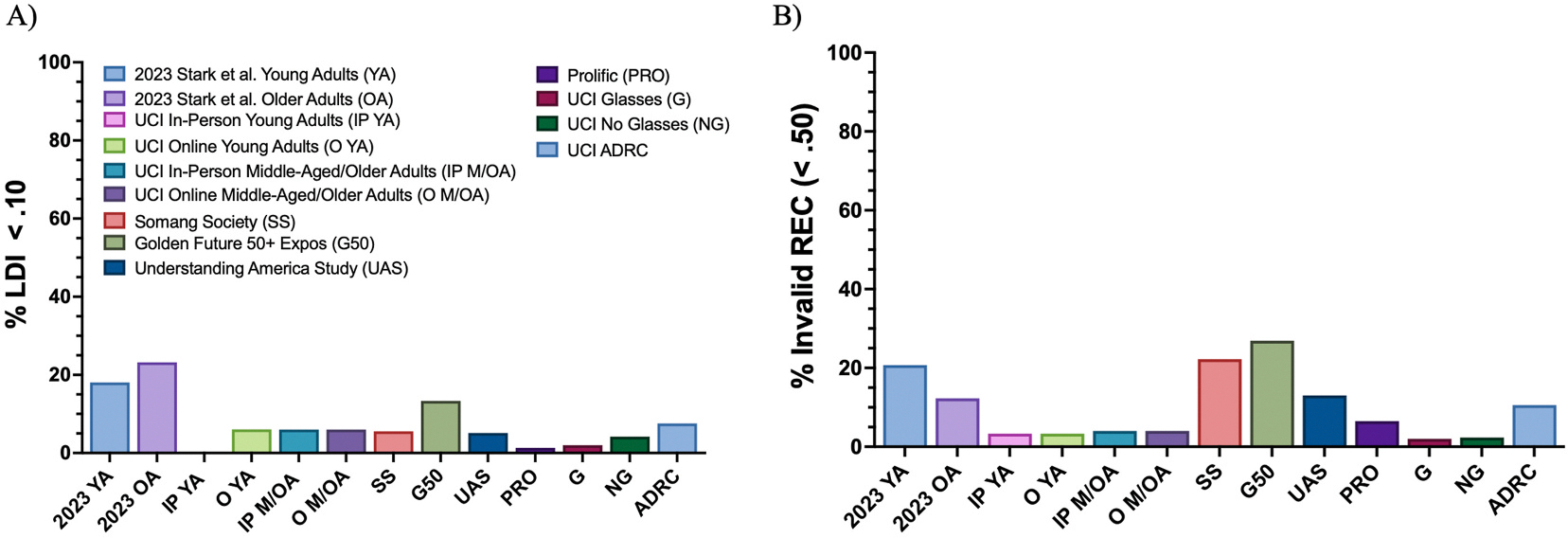
The percent of (A) LDI scores below .10 and (B) REC scores below .50 across experiments in comparison to the Stark et al. (2023) paper.

As noted, our previous testing has used a somewhat arbitrary threshold of 0.5 for a REC score to filter out participants who were noncompliant, misunderstood the instructions, or had serious memory issues (see Discussion). Using this, the adapted oMST from Stark et al. (2023) reported that 20.73 % of young adult data and 12.28 % of older adult data were rejected due to invalid REC scores using online testing. In comparison, the present study had an overall rate of 8.59 %. We did find some evidence for variation in rates of invalid REC scores across five experiments and their diverse settings (Fig. 6; Supplemental Fig. 1). In Experiment 1, 6.06 % of young adults had invalid REC scores in the online setting and 3.03 % had an invalid REC score in the in-person setting, while middle-aged and older adults had a 4.00 % invalid REC in each setting. Experiment 2’s outreach events showed that 22.22 % of adults from the Somang Society Korean Cultural Center and 26.92 % of participants from the Ontario, CA, and Long Beach, CA, Golden Future 50+ Senior Expos had invalid REC scores. In Experiment 3, 13.08 % of participants from the Understanding America Study and 6.21 % from Prolific were excluded due to invalid REC scores. In Experiment 4, 10.61 % participants recruited from the ADRC had invalid REC scores. Lastly, in Experiment 5, 2.00 % of participants in the Glasses condition and 2.08 % in the No Glasses condition had invalid REC scores. Thus, as one might expect, public outreach events resulted in higher levels of invalid REC scores than online or more private in-person testing.

To reiterate, participants were not excluded for having low LDI scores; however, we examined the proportion of individuals with LDI scores below 0.10 to identify those near floor-levels of performance, that would impair meaningful analyses. In Experiment 1, 6.06 % of young adults scored below 0.1 in the online setting, while none did so in the in-person setting. Among middle-aged and older adults, 4.00 % had LDI scores below .10 in the online setting and 6.00 % in the in person setting. In Experiment 2, 5.56 % of adults from the Somang Society Korean Cultural Center and 13.33 % of participants from the Ontario, CA, and Long Beach, CA, Golden Future 50+ Senior Expos had low LDI scores. Experiment 3 showed that 5.14 % of participants from the Understanding America Study and 1.30 % from Prolific had LDI scores below 0.10. In Experiment 4, 7.58 % of participants recruited from the ADRC had LDI scores below 0.10 (including both Dementia and No Dementia conditions). Finally, in Experiment 5, 2.00 % of participants in the Glasses condition and 4.17 % in the No Glasses condition scored below 0.10. Here again, the highest rates of exceptionally low performance came from the public outreach event.

## 4. Discussion

The present study tested the accessibility, reliability, and validity of the oMST (C. E. L. Stark et al., 2023), which was adapted from a popular research tool, the MST (Kirwan and Stark, 2007; S. M. Stark et al., 2019). The oMST is an automated task that can be self-administered or administered by a researcher/clinician. The task is automatically scored, generating two key outcome measures (LDI and REC) at the end of cognitive screening for use by researchers, clinicians, or respondents. Results from Experiments 1–5 observed subtle but reliable differences in lure discrimination across all age groups and testing sites. These results were also consistent across participant who were asked to complete the oMST with and without the use of their corrective lenses where there was no significant difference across LDI or REC scores between the two conditions. In fact, in our cross-experiment validation, we observed no significant differences in the slopes investigating age effects on LDI and REC across all the testing sites; further establishing the oMST as a reliable and accessible tool for cognitive screening across diverse testing environments and administration methods, addressing critical gaps in early screening for cognitive decline.

One concern when interpreting MST results and age-related effects in LDI is whether older adults can perceive the stimuli as well as younger adults, given normal age-related declines in visual acuity (Chen et al., 2017; Harrabi et al., 2015). Declines in sensory abilities, such as visual acuity, has previously been associated with declines in cognitive performance on neuropsychological cognitive screening batteries, such as the Mini Mental State Exam (Dearborn et al., 2018; Lin et al., 2004; Varadaraj et al., 2021). Additionally, Davidson et al. (2019) reported that caution should be taken when interpreting the LDI given that it was correlated with a visual perception composite score comprised from the Freiburg Visual Acuity Test (FRACT; Bach, 1996) and the VisTech Contrast Sensitivity Test. The composite score for these two visual perception tasks was created by combining their raw scores into an overall z-score. However, this method presents several challenges, including difficulties in interpreting the result and the implicit assumption that both tasks are weighed equally. There are fundamental differences across the two tasks assessing visual acuity, such that the FRACT primarily measures visual acuity (e.g., how well an individual can see fine details) while the VisTech Contrast Sensitivity Test assesses individuals’ ability to distinguish or see details in different levels of contrast.

In the present study, we directly manipulated visual acuity by recruiting participants who wear glasses or corrective lenses and having them complete four tasks designed to assess different aspects of visual acuity and perception (see Experiment 5). As confirmatory analyses, we used traditional measures of visual acuity, including the Tumbling E, Snellen, and MNREAD acuity charts. These analyses revealed that participants had difficulty completing the visual acuity tasks when not using their glasses or corrective lenses. In addition to these traditional measures, we included the sequential and simultaneous ICon, which assess visual acuity, object perception, working memory, and attention, using MST-like stimuli. We found that there was no significant difference between the glasses and no glasses conditions to successfully discriminate between similar lures. To further establish that reasonable ranges of visual acuity play a minor role in the ability to screen for dementia using the oMST, we asked the same group of participants to complete the task twice (counterbalanced), once with their glasses and once without. Once again, we found no significant difference in recognition memory or lure discrimination across all lure bins between the two conditions, suggesting that the age-related differences observed in LDI using the oMST are reliable and remarkably tolerant to modest changes in visual acuity. The manner in which and the degree to which stimuli change from target to lure is significant enough to make the difference trivial to spot in a pairwise comparison. Both with and without their glasses, participants made very few errors on the ICon tasks (Table 1). These are simple objects with overt visible changes, which is why the similarity index used across the lure bins is based on mnemonic confusion (incorrectly identifying the stimulus as *‘Old’* in an MST task, following a significant number of intervening trials) rather than a perceptual one. It’s not that participants can’t perceive the difference in color and features of the trombone shown in Fig. 1B, it’s that, given sufficient interference, those details did not remain in their memory.

While it is still possible that declining visual acuity can play a role on performance in cognitive screening batteries (and in the limit, it’s obvious it will), it is also possible the perception of the cognitive screening process can elicit poor performance. That is, the task may be perceived as jarring, and the complexity of some questions could be seen as demeaning if the respondent is unable to answer correctly, especially when a researcher is present during the screening (Krohne et al., 2011; Martin et al., 2015). This sense of embarrassment older adults reported during clinician-present cognitive screening assessments may even elicit a sense of increased subjective age (Geraci et al., 2018), which is associated with other aspects of cognition (i.e., psychological distress and mental health (Alonso Debreczeni and Bailey, 2021; Shrira et al., 2014);). However, this experience is alleviated when older adults are given the choice of when, where, and who can be present during the time of cognitive screening (Wong and Jacova, 2018). In fact, many older adults reported a preference of completing a cognitive screening measure alone at their home. The present study has demonstrated that the oMST is a versatile cognitive screening measure that can be completed in a research laboratory setting, during a community event with many bystanders present, or remotely using any device and location of choice, worldwide. This further strengthens its utility as an accurate tool for screening cognitive decline in environments that older adults perceive as safe and free from perceived judgment.

The oMST is a freely available web-based cognitive screening tool validated for global use on computers, tablets, and mobile devices. This fully automated, self-guided task requires approximately 5 min to complete, making it an efficient and user-friendly tool for cognitive screening. It is also automatically scored, calculating a recognition memory score and a lure discrimination index within seconds of task completion, which eliminates the need for manual scoring. Therefore, it significantly reduces the burden on clinicians and researchers, which is particularly important in underserved communities where access to cognitive screening may be limited. Furthermore, the automaticity of the oMST reduces uncertainty that may occur due to scoring and interpretation of the results. Additionally, the oMST has limited practice effects (C. E. L. Stark et al., 2023), allowing individuals to monitor their cognitive performance over time. This makes it an ideal tool for ongoing self-monitoring, allowing individuals to detect subtle changes and seek professional support if concerns arise. With its accessibility and reliability, the oMST can be reliability used as a cognitive screening tool anytime, anywhere. While the oMST the present study focused on evaluating the feasibility of administering the oMST across diverse testing contexts, it did not directly compare performance with other established cognitive screening tools, such as the Mini-Mental State Examination (MMSE) or Montreal Cognitive Assessment (MoCA). Future work should examine how the oMST’s accessibility and reliability in diverse contexts as relates to these traditional measures.

In previous studies, REC scores below the 0.50 threshold were typically considered invalid and excluded from data analysis. This was done to exclude participants who were not representative of the condition being studied. Even amongst older adults being tested online, this threshold can be > 4 standard deviations below the mean with a concurring large gap between these scores and the normal cluster. This can be seen to some extent in the long tails in the violin plots for REC in Fig. S3 and is even more apparent in the individual datapoints overlaid on these results in Fig. S1. Analyzing the distributions of REC scores for each test group (Fig. S2) shows a common main group of participants with a high REC score (0.8–0.9) and other separate low-performance groups forming variable tails on the left side of the distributions.

We’ve taken this to represent some combination of non-compliance, misunderstanding of the instructions, or potential clinical impairment that makes the participants non-representative of the targeted healthy population. In addition, however, low REC scores may indicate that participants respond more conservatively, with a bias towards fewer *‘Old’* responses, despite compliance and no actual impairment to their memory. At times, we do observe “invalid” REC scores in the face of reasonable LDI scores on the same test. Given that the present study aimed to validate the use of the oMST across diverse testing sites, we feel it was reasonable here to exclude participants with REC scores below this threshold. However, we do not suggest that this is the ideal approach. Not only would it remove those suffering from dementia (when this may be a target population to identify), but it does suffer from that concern surrounding response bias. Traditional signal detection’s criterion bias metric unfortunately cannot be used (given that there are three response options that are not necessarily ordinal), but other approaches do exist. In prior work, we have used a Bayesian cognitive model to successfully identify those who were guessing (Lee and Stark, 2023). Whether that, or more traditional measures based on reaction time or response rates is most effective will need to be explored in future work. We acknowledge that the removal of REC scores below 0.50 is provisional, and future work should apply cognitive modeling to cluster participants with low REC scores (e.g., random responders, guessers, and poor performers) to provide more guidance on exclusion thresholds. However, we also note that the overall rate here was relatively low at 8.59 % and that the results were quite similar when the entire populations were analyzed (Supplemental Materials; Supplementary Table 2).

Building on these considerations regarding exclusion criteria, it is also important to examine how exclusion rates varied across different testing contexts, particularly those that may have higher proportions of impaired individuals. Across all experiments, participants with low REC scores were excluded to ensure data quality, though this may limit generalizability to populations with more severe cognitive impairments. The present study, however, aimed to assess the general feasibility, reliability, and accessibility of the oMST across diverse contexts, including lab-based, remote, and community-based settings. Notably, exclusion rates were higher in the community-based setting compared with online and in-lab testing. Several factors likely contributed to these differences, including reduced task motivation, environmental distractions, and variability in participants’ understanding of task instructions in less controlled environments. Participants with REC scores below 0.50 were excluded from analyses based on prior work showing that even older adults with mild cognitive impairment typically achieve REC scores of ~0.60 (Stark et al., 2013). Scores below 0.50 are therefore atypical, even for clinical populations, and may reflect lack of motivation, random button pressing, or guessing rather than poor memory ability. Consistent with this, item-level inspection of the present data indicated that participants with REC <0.50, particularly healthy young adults, often engaged in random responding rather than meaningful task completion. The REC cutoff helps reduce noise in the data without broadly excluding participants. These observations highlight the reliability, accessibility, and feasibility of the oMST, while also noting that certain populations or testing environments may present challenges affecting data quality.

We would, however, offer a note of caution regarding the use of the oMST in outreach settings. If further data collection is possible, we recommend that the oMST be readministered in a controlled laboratory environment. This is particularly important given the noisier nature of data obtained in real-world community events, where environmental distractions and variability in engagement can affect data quality. The oMST is certainly not immune to these effects and the community-based setting yielded the highest exclusion rates due to REC scores below 0.50. Such community-based testing can, however, be viably used for screening, population enrichment, and recruitment.

### 4.1. Limitations

While the present study demonstrated the feasibility, reliability, and validity of the oMST across diverse testing contexts, several limitations should be noted. First, ceiling effects were observed in REC and LDI scores for young, healthy adults, which may reduce sensitivity in detecting subtle cognitive changes in this population. However, the oMST is sensitive to hippocampal-dependent cognitive changes, which are unlikely to be present in younger adults, and our results demonstrate robust performance across lab-based, remote, and community-based settings. Second, this study did not directly compare oMST performance with other established cognitive screening tools, such as the Mini-Mental State Examination (MMSE) or Montreal Cognitive Assessment (MoCA). Future work should examine how the oMST’s accessibility and reliability relate to these traditional measures to further establish its clinical utility. Third, potential confounding factors such as attention, motivation, or technological proficiency, particularly in remote or community-based testing settings, could have influenced performance. While visual acuity was controlled, future studies should systematically evaluate these factors to further ensure the reliability of the oMST in real-world contexts. Fourth, differences in exclusion rates across settings may limit generalizability. Across all experiments, participants with low REC scores were excluded to ensure data quality. Exclusion rates were notably higher in community-based settings compared with online and in-lab samples, likely due to factors such as reduced motivation, environmental distractions, and variability in task comprehension.

### 4.2. Conclusion

The present study aimed to validate the oMST as an accessible and reliable tool for cognitive screening in diverse settings, or ‘in the wild.’ We found that, regardless of testing site or modality, age effects observed on LDI were consistent and robust. Specifically, age-related declines in visual acuity did not significantly affect participants’ recognition memory performance or ability to discriminate between similar lures. This suggests that the oMST can effectively be used across a wide range of age groups and settings, supporting its utility as a versatile tool for cognitive screening and population enrichment in any environment.

## Supplementary Material

1

## Figures and Tables

**Table 1:** Means, SD, and t-test statistics for the Glasses and No Glasses conditions across all tests.

	Glasses		No Glasses			
				
	*M*	*SD*	*M*	*SD*	*t*	*p*

Tumbling E	93.22	13.59	82.95	20.73	3.82	<.001***
Snellen	1.22	0.49	0.61	0.39	6.03	<.001***
MNREAD Acuity Chart	0.59	0.27	1.23	1.08	3.67	<.001***
Sequential ICon	2.71	0.69	2.62	0.82	0.76	.453
Simultaneous ICon	2.77	0.62	2.86	0.48	1.20	.238
LDI	0.56	0.20	0.52	0.18	1.54	.132
REC	0.87	0.11	0.88	0.09	0.39	.697

Note.

* p < .05

** p < .01

*** p < .001.

**Table 2:** Demographic data for Experiment 3 demonstrating the diversity of the sample and illustrating the feasibility of administering the oMST remotely across diverse contexts.

Experiment	Country of Residence	Region of Residence

1	100 % United States	100 % West
2	100 % United States	100 % West
3a	100 % United States	39.13 % South
		26.94 % Midwest
		18.94 % Northeast
		13.24 % West
		1.83 % Plains
3b	18.46 % Mexico	
	10.46 % Canada	
	8.62 % Italy	
	7.69 % Greece	
	7.38 % Hungary	
	6.15 % Germany	
	4.92 % Kenya	
	2.77 % Chile	
	2.15 % France	
	1.85 % Belgium	
	1.85 % Brazil	
	1.85 % China	
	1.85 % Czech Republic	
	1.85 % India	
	1.85 % Philippines	
	1.54 % Austria	
	1.54 % Israel	
	1.23 % Bangladesh	
	1.23 % Croatia	
	1.23 % Finland	
	1.23 % Netherlands	
	1.23 % Nigeria	
	0.92 % Egypt	
4	100 % United States	100 % West
5	100 % United States	100 % West

*Note*. Data reported from Experiment 3a is represents the Understanding America Study participants. Region of residence for the United States is represented by the South (Virginia, Georgia, Florida, North Carolina, South Carolina, Tennessee, Alabama, Louisiana, Texas, Arkansas, Mississippi, West Virginia, District of Columbia), Midwest (Ohio, Michigan, Indiana, Illinois, Wisconsin, Missouri, Minnesota, Iowa, Kansas), Northeast (Pennsylvania, New York, New Jersey, Massachusetts, Connecticut, Maine, New Hampshire), West (California, Washington, Oregon, Montana, Nevada, Colorado, Hawaii, Arizona), and the Plains (South Dakota, Oklahoma). Data reported from Experiment 3b represents participants recruited from Prolific. This table reports the ethnic/race break-down of participants across experiments. The purpose of including these data is to illustrate the diversity of the sample and the feasibility of administering the oMST across varied participants and community settings. These percentages are provided for descriptive purposes only. No analyses of ethnic differences in oMST performance were conducted.

**Table 3:** oMST primary outcome measures (LDI and REC) across the different experiments.

Testing Site		LDI		REC	
			
	*n*	*r*	*p*	*r*	*p*

UCI In-Person	77	−.50	<.001***	−.24	.036*
UCI Online	77	−.42	<.001***	−.09	.430
Golden Future Senior 50+ Expo	43	−.14	.359	−.23	.140
Understanding America Study	186	−.36	<.001***	−.16	.031*
Prolific	937	−.02	.605	−.02	.502
UCI ADRC – No Dementia	109	−.23	.017*	−.09	.373
UCI - Glasses	46	−.62	<.001***	−.49	<.001***
UCI – No Glasses	46	−.51	<.001***	−.51	<.001***

*Note*. Correlations between Age for LDI and REC.

* p < .05

** p < .01

*** p < .001.

## Data Availability

Data is set to private for review and will be made available on Dryad on Acceptance.

## Appendix A. Supplementary data

Supplementary data to this article can be found online at https://doi.org/10.1016/j.neuropsychologia.2025.109341.
