# Supplementary material for: MST in the wild: Optimizing the mnemonic similarity task for use in diverse environments
Source: Neuropsychologia. Author manuscript; Available in PMC 2026 May 26. (PMC13203989; doi:10.1016/j.neuropsychologia.2025.109341)
Supplement: 1 [file NIHMS2168629-supplement-1.docx]

**Supplementary Data and Analyses**

While the analyses in the main text includes only participants with REC scores at or above .50, the current analyses reports the primary comparisons across experiments when including all participants regardless of REC scores are included. Specifically, we compared data from Experiment 1 (UCI in-lab or remote) with each dataset collected using the same modality of oMST administration. In Experiment 2, we ran two separate analyses comparing community outreach participants with age-matched in-person participants at UCI. When including all data, we observed a reliable decrease of 0.14 (0.59 SD) in LDI scores between the community outreach events (*M* = .39, *SD* = .22) and the in-person lab sample (*M* = .53, *SD* = .20), *t* (121) = 3.24, *p* < .001. We also found a reliable decrease of 0.17 (0.74 SD) in REC scores between the community outreach events (*M* = .64, *SD* = .26) and in-person lab data (*M* = .81, *SD* = .14), *t* (121) = 4.12, *p* < .001.

In Experiment 3 we compared age-matched data from Experiment 1 with UAS data. As in the original analyses, a t-test indicated a significant difference in LDI, with lower scores in UAS (*M* = .44, *SD* = .21) than in UCI online (*M* = .54, *SD* = .20), *t* (262) = 3.21, *p* = .001. A Mann–Whitney test again showed no significant difference in REC (UAS: Mdn = .80, *M* = .73, *SD* = .23; UCI online: Mdn = .78, *M* = .77, *SD* = .17), U = 5019, *p* = .496. We also compared age-matched data from Experiment 1 with Prolific data. A t-test indicated a significant difference in LDI (Prolific: *M* = .60, *SD* = .18; UCI online: *M* = .68, *SD* = .24), *t* (1028) = 2.43, *p* = .015. Unlike in the original manuscript, when including all REC scores, the Mann–Whitney test showed no significant difference in REC (Prolific: Mdn = .85, *M* = .82, *SD* = .20; UCI online: Mdn = .85, *M* = .79, *SD* = .20), U = 13464, *p* = .215.

In Experiment 4 we compared oMST performance in ADRC participants scoring 0 (No Dementia) on the CDR with age-matched participants from Experiment 1. As before, there was no significant mean difference in LDI (ADRC: *M* = .46, *SD* = .20; in-person lab: *M* = .52, *SD* = .20), *t* (162) = 1.81, *p* = .072. Similarly, a Mann–Whitney test showed no significant difference in REC (ADRC: Mdn = .80, *M* = .75, *SD* = .19; in-person lab: Mdn = .78, *M* = .79, *SD* = .14), U = 2461, *p* = .426.

Overall, the reanalysis of data including all REC scores yielded the same overall pattern of results as those reported in the main text when excluding participants with REC < .50. These findings demonstrate that the key conclusions are not dependent on this exclusion criterion and remain robust across analytic approaches. At the same time, we maintain that restricting analyses to REC ≥ .50 provides the most valid assessment of task performance by minimizing the influence of task disengaged, guessing, or random responding.

**Supplementary Table 1. Overview of Experiments and Testing Conditions**

| **Experiment** | **N** | **Modality** | **Testing Context** |
| --- | --- | --- | --- |
| Exp. 1 | 77 | In-person and Remote (web-based) | UCI lab vs Remote |
| Exp. 2 | 58 | Community-based in-person testing | Golden Future 50+ Senior Expos and the Somang Society Korean Cultural Center |
| Exp. 3 | 1,123 | Remote (web-based) | Understanding America Study and Prolific online global testing platform |
| Exp. 4 | 265 | In-person | UCI ADRC |
| Exp. 5 | 46 | In-person | UCI lab; Glasses vs No Glasses |

*Note.* N is the total number of participants used in the final analyses.

**Supplementary Table 2. All REC Scores Reanalysis of Data**

| Experiment | *t* _(LDI_*_)_* | *p _(_*_LDI)_ | *t* / Mann–Whitney U _(REC)_ | *p* _(REC)_ |
| --- | --- | --- | --- | --- |
| 2 (Community Outreach) | 3.24 | < .001 | 4.12 | < .001 |
| 3a (UAS) | 3.21 | .001 | 5019 | .496 |
| 3b (Prolific) | 2.43 | .015 | 13464 | .215 |
| 4 (ADRC ND) | 1.81 | .072 | 2461 | .426 |

*Note*. This table presents a reanalysis including all participants across all REC scores compared against the age-matched and testing-mode samples from Experiment 1. The overall pattern of results is consistent with the original analyses that excluded participants with REC scores below 0.50.

**Supplementary Figure 1:**


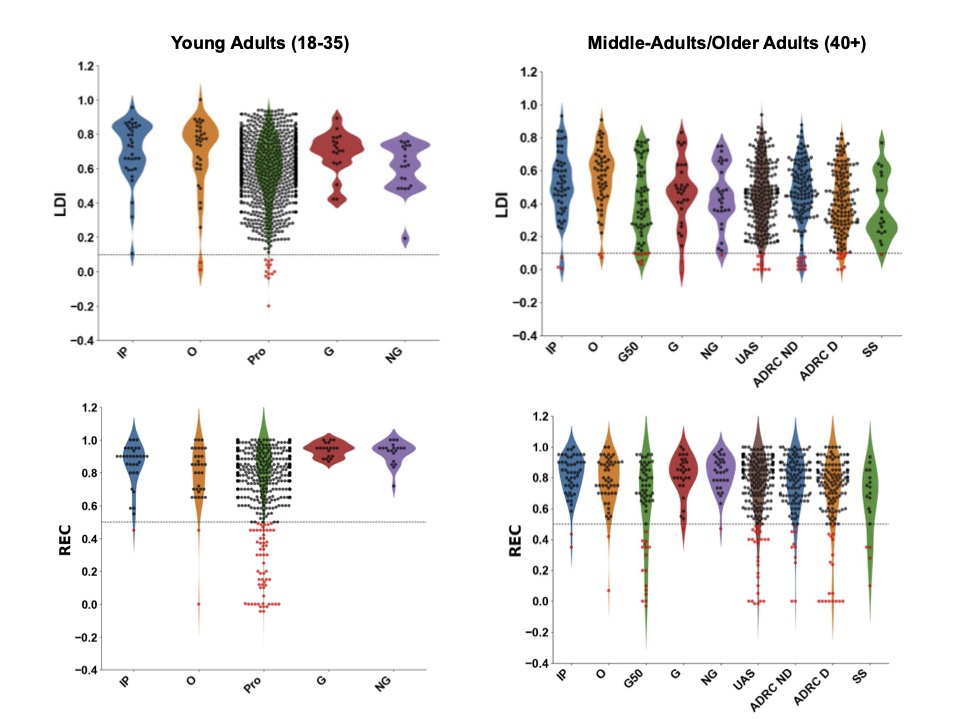


Figure S1: Paralleling Figure 3, but indicating individual participant data.

**Supplementary Figure 2:**


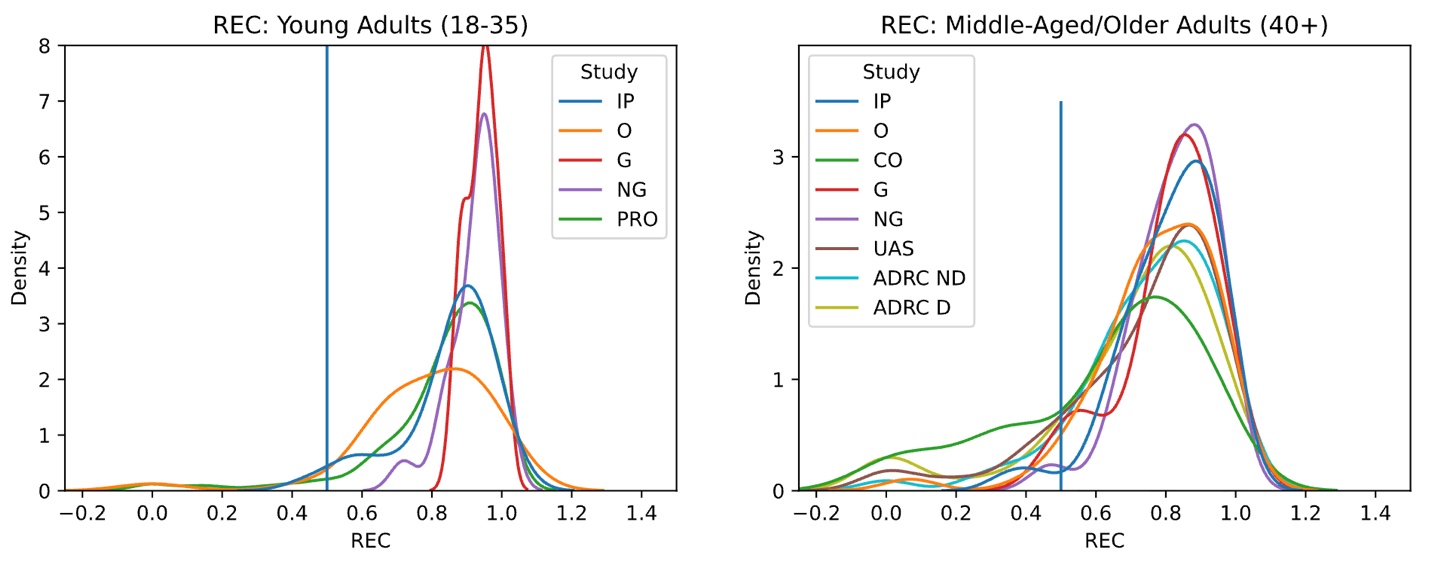


Figure S2: Distribution of REC scores for all experimental conditions split by age groups. The vertical line at 0.5 shows the REC threshold used to isolate data likely to be contaminated. Note in each distribution a clear main component with a mean REC of 0.8 – 0.9 and evidence of one or more other components leading to the leftward skew or bimodal features. The REC threshold was designed to isolate exceptionally low performance.

**Supplementary Figure 3:**


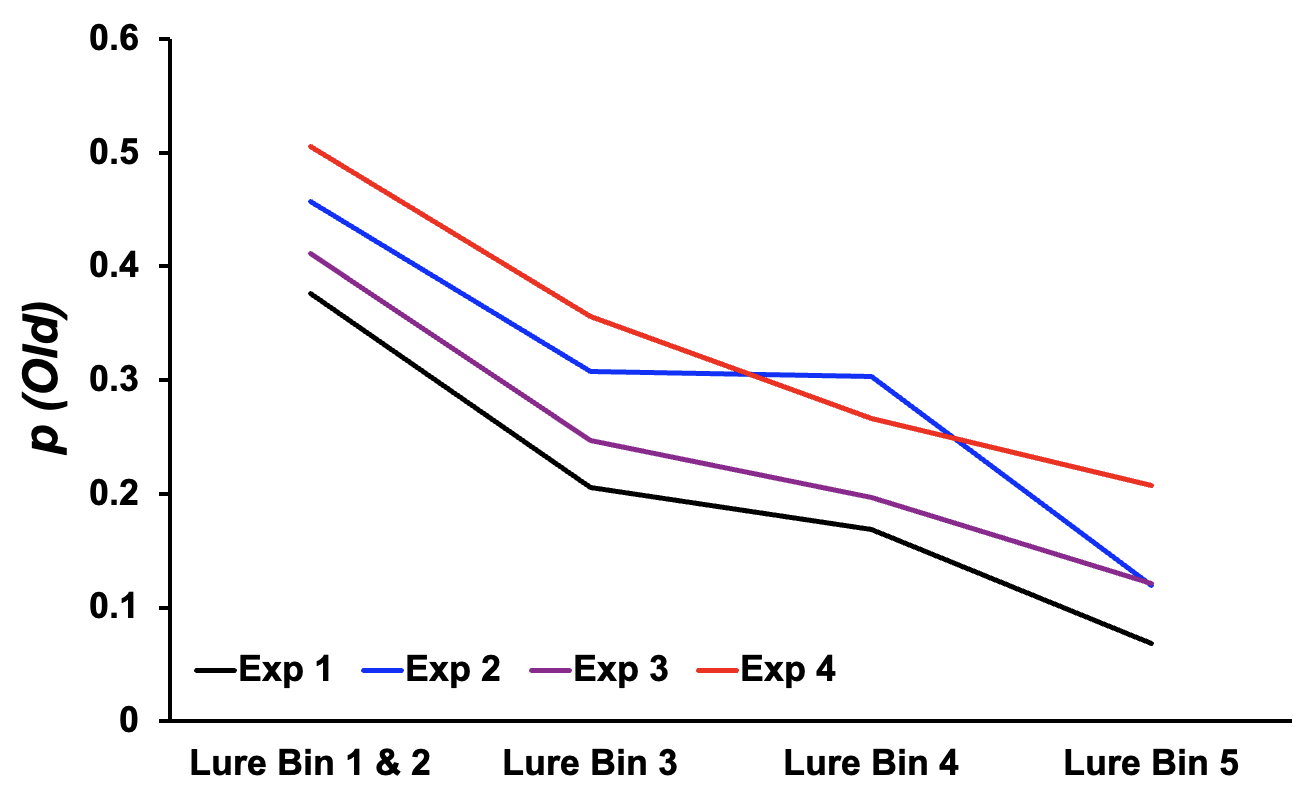


Figure S3: Paralleling Figure 4, performance across lure bins for each experiment. As expected with the oMST, stimuli in the lower lure bins are more likely to be mistakenly judged as “old”. Stimuli from the MST and oMST have been previously measured, item by item, in terms of their “mnemonic similarity” by assessing this measure and binning stimuli into difficulty groups.

**Supplementary Figure 4:**


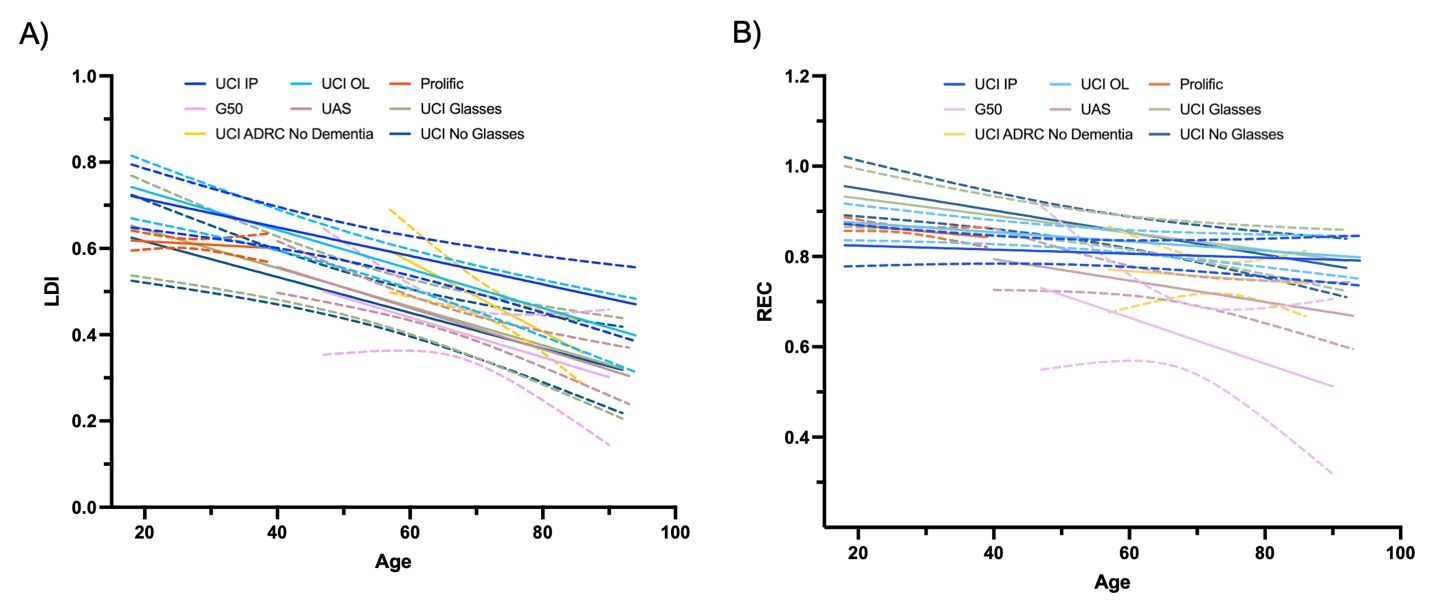


Figure S4: Regression analyses examining age-effects across (A) LDI and (B) REC for the different testing contexts with confidence intervals.
